# Propagation of negative shocks across nation-wide firm networks

**DOI:** 10.1371/journal.pone.0213648

**Published:** 2019-03-14

**Authors:** Hiroyasu Inoue, Yasuyuki Todo

**Affiliations:** 1 Graduate School of Simulation Studies, University of Hyogo, Kobe, Japan; 2 Graduate School of Economics, Waseda University, Tokyo, Japan; 3 Research Institute of Economy, Trade, and Industry, Tokyo, Japan; Nagoya University, JAPAN

## Abstract

This study examines how negative shocks due to, for example, natural disasters propagate through supply chains. We apply a simulation technique to actual supply chain data covering most Japanese firms. To investigate the property of the propagation in the network, we test different types of artificial negative shocks. We find that, first, network structures severely affect the speed of propagation in the short run, and the total loss in the long run. The scale-free nature of the actual supply-chain network—that is, the power-law degree distribution—leads to faster propagation. Second, more intensive damages—that is, more damages suffered by fewer firms—result in faster propagation than extensive damages of the same total size. Third, the actual supply-chain network has innate robustness that comes from substitutability of supplies. If the supply-chain network has severe substitutability, the propagation of negative shocks becomes substantially large. Fourth, direct damages in urban regions promote faster propagation than those in rural regions. Fifth, different sectoral damages show significant differences in the speed of propagation. Finally, we check the indirect damage triggered by a single firm’s loss: 9.7% of all firms contribute to significant loss, and this loss accounts for more than 10% of the damage to the entire production. The simulations conspicuously show that different direct damages, even if they have the same total magnitude of damages, can generate considerably different damages because of the structure of the supply-chain network.

## Introduction

Natural disasters trigger economic damages directly and indirectly because negative shocks are largely propagated through supply chains [1]. These indirect damages are far from negligible; they often constitute a large share of total damages [2, 3]. For example, let us consider the aftermath of the Great East Japan Earthquake in 2011. Although many firms, including those in foreign countries, were directly unaffected by the earthquake, they were compelled to cease operations due to supply shortages.

It is important to know how and to what extent negative shocks propagate in the economy through supply chains. Different economic models have been used to quantitatively examine these aspects [4]. One major approach is based on input-output (IO) tables [5], which are matrices depicting input-output relationships among different sectors in the economy. IO tables show the amount of inputs from each sector for producing a dollar’s worth of production in a sector. These tables have been used to estimate the effects of a shock in a sector on the production in other sectors through inter-sectoral production relationships [6, 7]. Okuyama et al. particularly examined the indirect effects of natural disasters because of supply chain disruptions using IO tables [8]. Although earlier studies relied on fixed IO tables before and after disasters, Rose and Liao incorporated flexible coefficients by employing computable general equilibrium (CGE) models [9]. In their model, the amount of inputs required for production in a particular sector is endogenously determined in the model, and thus can be changed after a shock.

However, IO tables cannot fully aid us in examining the propagation of negative shocks in supply chains because they only capture production relationships at the sectoral level and not at the firm level [6]. That is, how indirect damages propagate through supply chains heavily depends on how directly damaged firms are connected with other firms [10]. However, IO tables do not capture these networks at the firm level.

To overcome the shortcoming of models based on IO tables, Hallegatte proposed an agent-based model wherein firms, rather than sectors, interact with each other through supply chains [10]. Using the model, they simulated the propagation of negative shock through supply chains. Although they incorporated actual IO relationships at the sectoral level into the model, they relied on hypothetical random networks because they lacked actual data on supply chain relationships among firms. On the other hand, Bak et al. and Delli et al. utilized firm-level models, but did not incorporate actual data [11, 12].

The use of hypothetical networks, rather than actual ones, in simulation analysis is a drawback. This is because the literature on network science shows that differences in the network structure can lead to a substantial difference in behaviors of agents in the network [13, 14]. Recent economics literature also focuses on the role of the structure of firm networks in the propagation of negative shocks [15, 16]. Therefore, one may not obtain reasonable conclusions about how damages propagate through supply chains without using the actual data on the networks of firms in an economy. This kind of investigation is scarce, although propagation of negative shocks through financial networks of firms has been modeled [17, 18] and empirically examined using actual data [19, 20].

Using the same data and model as the current study, a prior study estimated the indirect damages caused by the Great East Japan Earthquake and the predicted Nankai Trough Earthquake [21], which are actual and expected actual disasters, respectively. On the other hand, the vulnerability and robustness of the economic system have not been comprehensively studied until now. We thus reveal the intrinsic nature of the properties of the system by testing different comprehensive sets of artificial damages as virtual disasters.

We utilize the nation-wide supply chain data in Japan and a modified model of [10] to tackle this issue. By simulating supply chains using different assumptions, we can address the following important issues. First, by comparing outcomes of actual networks with hypothetical ones used in extant literature, we highlight the importance of the network structure in the propagation of shocks through supply chains. Second, we examine how different intensities of direct damages lead to different indirect damages through supply chains. We find that intensive damages—that is, larger damages suffered by fewer firms—result in faster propagation than extensive damages—that is, smaller damages suffered by more firms—of the same total amount of initial direct damages. Third, to highlight the importance of substitution of suppliers in the wake of supply chain disruption, we compare the benchmark case with cases wherein substitution is more restricted. We find a significant role of substitution in mitigating propagation here. Fourth, we examine how direct damages in different regions affect the propagation pattern. This analysis reveals that direct damages in industrial areas result in faster propagation than those in remote areas. However, the total amount of damages in the long run is the same. Fifth, the effects of direct damages in different sectors are also explored. Direct damages in sectors for which supply chains are regionally clustered—most notably the construction sector—lead to small indirect damages. Finally, the estimation of indirect damages triggered by a single firm’s loss shows that 9.7% of all firms contribute to substantially large indirect damage. All these results suggest that the propagation of negative shocks and total damages due to disasters largely depends on the structure of the supply chain network in an economy.

## Data

We use two databases collected in 2011 by Tokyo Shoko Research (TSR) (one of the two major corporate research companies in Japan)—the TSR Company Information Database and the TSR Company Linkage Database. The databases are commercially available. In our case, we have access to the databases that are licensed to the Research Institute of Economy, Trade and Industry (RIETI). The TSR data contain a wide range of firm information, including identification numbers of suppliers and clients of each firm. Although the maximum number of suppliers and clients reported by each firm is 24, we can capture more than 24 suppliers and clients by looking at the supplier-client relationships in the reverse direction. In other words, although a large firm such as Toyota reports only 24 suppliers, its suppliers are most likely to report Toyota as their client. Accordingly, we identify the supply chain network of firms in Japan to a great extent. The number of firms or nodes is 1,109,549, whereas the number of supplier-client ties or links is 5,106,081. This network is directed as it represents the flow of intermediate and final products.

Firms are classified into sectors or industries. In the TSR data, industries are categorized according to the Japan Standard Industrial Classification (JSIC) [22]. The 1,460 classifications at the four-digit level of JSIC are converted into the 190 basic sector classifications of the IO tables because we will later incorporate IO tables into the firm-level data.

Although the TSR data contain information about suppliers and clients, they do not include information on the value of transactions in each supplier-client link. We conduct the following two-step calculations to estimate the transaction value. First, each supplier’s sales are divided into its clients’ purchases by using the clients’ sales as weights. This step provides each link a transaction value. The transaction values at the tie-level from the first step can be aggregated into transaction values at the sector level, that is, from each sector to another. The sector-level transaction values, however, may not match those from the IO tables. This inconsistency between micro and macro data comes from the incompleteness of the TSR data, which may not cover all firms in the economy. Additionally, the TSR data do not capture transaction ties with final consumers. Therefore, in the second step, we modify the transaction values at the tie-level by using the IO tables for Japan in 2011 [23]. Specifically, the value of sector A sold to sector B (taken from the IO tables) is proportionally divided among all ties between the suppliers in sector A and clients in sector B which is obtained from their tie-level transaction values. Similarly, the value of the final consumption of sector A in the IO tables is proportionally divided among all suppliers in sector A. Through these steps, the aggregate tie-level transaction values equal those from the IO tables. Thus, the value of damages estimated from our simulation can be reasonably compared with macroeconomic statistics, such as GDP.

## Theoretical model

We utilize the theoretical model proposed by [10] with some modifications. As we will indicate later, the major difference is in the rationing mechanism. Moreover, the target inventory size has a Poisson distribution, instead of a common constant. This is an agent-based model wherein agents, that is, firms and final consumers, follow specific rules. In the model, each firm in a sector produces a sector-specific product using a variety of intermediates, and delivers the product to its clients and final consumers. Further, we assume that firms have inventories of intermediates for dealing with a possible supply shortage.

We will later consider a situation wherein an artificial disaster hits some firms, and the damages from the disaster affect the production. Then, this makes it difficult for the firm to meet the demand. However, let us first describe a pre-disaster situation. The daily trade volume from supplier *j* to client *i* before the disaster is denoted by *A*_*i*,*j*_, whereas the daily trade volume from firm *i* to final consumers is denoted by *C*_*i*_. Subsequently, the initial production of firm *i* on the day before the disaster is

$$\begin{array}{c} P_{\text{ini}i}=\Sigma_{j}A_{j,i}+C_{i}. \end{array}$$ (1)

We further assume that firm *i* has an inventory of *S*_*i*,*j*_ of the intermediate good produced by firm *j*, and restores the inventory to a level equal to a given number of days *n*_*i*_ for the utilization of the product supplied by firm *j*. On day *t*, the orders from firm *i* to its supplier *j*, denoted by *O*_*i*,*j*_(*t*), is given by

$$\begin{array}{c} O_{i,j}\left(t\right)=A_{i,j}\frac{D_{i}^{*}\left(t-1\right)}{P_{\text{ini}i}}+\frac{1}{\tau }\left(n_{i}A_{i,j}\frac{D_{i}^{*}\left(t-1\right)}{P_{\text{ini}i}}-S_{i,j}\left(t\right)\right), \end{array}$$ (2)

where $D_{i}^{*}\left(t-1\right)$ is a realized demand for firm *i* on day *t* − 1, the previous day; and *τ* is the number of days or the rate to adjust its inventory size. For example, when *τ* is six, as we assume in our simulations, the firms plan to fill the gap between the projected inventory (i.e., *n*_*i*_ days of demand) and the actual inventory gradually by the rate of one over six. It must be noted that *n*_*i*_ is generated from the Poisson distribution for each firm in the beginning of every simulation. The first term in the right-hand side of Eq (2) is the amount of product supplied by firm *j* that is needed to satisfy the demand on the previous day. The second term indicates the amount of the intermediate good by firm *j* that is needed to restore the inventory to the projected level. Accordingly, the total demand for product of firm *i* on day *t*, *D*_*i*_(*t*), is given by the sum of the final demand from consumers and the total orders from its clients:

$$\begin{array}{c} D_{i}\left(t\right)=\Sigma_{j}O_{j,i}\left(t\right)+C_{i}. \end{array}$$ (3)

Now, suppose a disaster hits the economy and damages firm *i* directly. We assume that a certain proportion, *δ*_*i*_, of production capacity of firm *i* is destroyed by the disaster. Subsequently, the production capacity of firm *i* is denoted by *P*_cap*i*_. It can be considered the maximum production, assuming that there is no supply shortage.

$$\begin{array}{c} P_{{\text{cap}}_{i}}=P_{\text{ini}i}\left(1-\delta_{i}\right). \end{array}$$ (4)

The production of firms may also be limited by a supply shortage. Since we assume that firms in the same sector produce the same product, the supply shortage from firm *j* in sector *s* can be compensated by supplies from firm *k* in the same sector if firm *i* is a client of *j* and *k*. We do not assume changes in supply chain ties after the disaster. Subsequently, the total inventories of product *s* in firm *i* on day *t* can be indicated as

$$\begin{array}{c} S_{\text{tot}i,s}\left(t\right)=\Sigma_{j\in s}S_{i,j}\left(t\right). \end{array}$$ (5)

The initial consumption of product *s* at firm *i* is also defined for convenience.

$$\begin{array}{c} A_{\text{tot}i,s}=\Sigma_{j\in s}A_{i,j}. \end{array}$$ (6)

Using the above two variables, *P*_pro*i*,*s*_(*t*), the maximum production for firm *i* limited due to the inventory of product *s* on day *t* is obtained as follows.

$$\begin{array}{c} P_{\text{pro}i,s}\left(t\right)=\frac{S_{\text{tot}i,s}\left(t\right)}{A_{\text{tot}i,s}}P_{\text{ini}i}. \end{array}$$ (7)

Now, we can determine the maximum production of firm *i* on day *t*, considering its production capacity, *P*_cap*i*_, and its production constraints due to shortage of supplies, *P*_pro*i*,*s*_(*t*):

$$\begin{array}{c} P_{\text{max}i}\left(t\right)=\text{Min}(P_{\text{cap}i},{\text{Min}}_{s}\left(P_{\text{pro}i,s}\left(t\right)\right). \end{array}$$ (8)

Therefore, the actual production of firm *i* on day *t* is

$$\begin{array}{c} P_{\text{act}i}\left(t\right)=\text{Min}\left(P_{\text{max}i}\left(t\right),D_{i}\left(t\right)\right) \end{array}$$ (9)

When the demand for a firm is greater than its production capacity, the firm cannot completely satisfy its demand, as shown in Eq (9). In this case, firms should ration their production to their clients. Hallegatte proposed a rationing policy where each client and the final consumer would receive an amount of product that is the same fraction (*P*_act*i*_/*P*_ini*i*_) as its pre-disaster trade volume [10]. However, this may not be the case in practice. Suppose there is a client *h* and supplier *i* in sector *r*; supplier *i* supplies to all the clients normally. Client *h* increases its demand for product *r* because other suppliers of the product *r* for the client *h* reduce their supply as a result of the disaster. Then, according to this rationing policy, supplier *i* is compelled to decrease the supply of product *r* to other clients, even if it is not affected by the disaster—this should ideally not occur. Thus, this rationing policy is most likely to augment the propagation of negative shocks, leading to an overvaluation of the effects of disasters. For example, 10% of the damages (*δ* = 0.1) suffered by a few firms (such as a 100 randomly selected firms) can make the entire supply chain network ineffective, which may not happen in an actual economy.

Therefore, we present a rationing policy wherein firms are prioritized according to the level of order after the disaster to their initial order. Importantly, this proposed rationing policy seems to be reasonable because this policy is equal for clients.

The policy is described as follows: notably, we do not have to consider a rationing policy if the production capacity is larger than the demand. We use the same notation as above. Here, a firm *i* has clients *j* and a final consumer. We can calculate the ratio of the order after and before the disaster. This is notated as $O_{j,i}^{rel}$ for customers and $O_{c}^{rel}$ for a final consumer. $O_{j,i}^{sub}$ and $O_{c}^{sub}$ are temporal variables to calculate the realized order. They are initialized by zero. The calculation follows the steps outlined below.

1. Obtain the remaining production *r* of firm *i*
2. Calculate $O_{\text{min}}^{rel}=\text{Min}\left(O_{j,i}^{rel},O_{c}^{rel}\right)$
3. If $r\leq (\sum_{j}O_{\text{min}}^{rel}O_{j,i}+O_{\text{min}}^{rel}C_{i})$ then proceed to 8
4. Add $O_{\text{min}}^{rel}$ to $O_{j,i}^{sub}$ and $O_{c}^{sub}$
5. Subtract $\left(\sum_{j}O_{\text{min}}^{rel}O_{j,i}+O_{\text{min}}^{rel}C_{i}\right)$ from *r*
6. Remove the client or the final consumer that indicated $O_{\text{min}}^{rel}$ from the calculation
7. Back to 2
8. Calculate *O*^*rea*^ that satisfies *r* = (∑_*j*_ *O*^*rea*^ *O*_*j*,*i*_ + *O*^*rea*^ *C*_*i*_)
9. Get $O_{j,i}^{*}=O^{rea}O_{j,i}+O_{j,i}^{sub}O_{j,i}$ and $C_{i}^{*}=O^{rea}C_{i}+O_{c}^{sub}C_{i}$ where the realized order from firm *j* to supplier *i* is denoted as $O_{j,i}^{*}\left(t\right)$, and the realized order from a final consumer is $C_{i}^{*}$
10. Finalize the calculation

Following the rationing policy, the realized total demand for firm *i*, $D_{i}^{*}\left(t\right)$, is

$$\begin{array}{c} D_{i}^{*}\left(t\right)=\Sigma_{j}O_{j,i}^{*}\left(t\right)+C_{i}^{*}. \end{array}$$ (10)

Accordingly, the inventory of firm *j*’s product in firm *i* is renewed by

$$\begin{array}{c} S_{i,j}\left(t+1\right)=S_{i,j}\left(t\right)+O_{i,j}^{*}\left(t\right)-A_{i,j}\frac{P_{\text{act}i}\left(t\right)}{P_{\text{ini}i}}. \end{array}$$ (11)

Using the model above, we simulate how direct damages, represented by an exogenous reduction in the production capacity of a set of firms, affect the production of the entire economy through the propagation of negative shocks along supply chains. In the simulation, we use the actual supply chains of firms in Japan based on the TSR data. *A*_*i*,*j*_ and *C*_*i*_ are determined using the IO tables and supply chain ties, as described in the section “Data.” We assume that *τ* is 6 as per [10]; the setting of the parameter is ad hoc, although it is desirable for the setting to be supported by empirical data. At the beginning of the simulation, *n*_*i*_ is assigned to each firm from the Poisson distribution with a mean of 15. This parameter also requires empirical support, if it is possible.

In each simulation, exogenous damages are given on day 0. Specifically, our benchmark simulation assumes that 10,000 firms that are randomly selected from 1,109,549 firms lose 50% of their production capacity after the disaster (i.e., *δ*_*i*_ in Eq (4) is 0.5), although we experiment with other types of shock (explained later). In other words, the benchmark cases assume that approximately 0.5% (10,000 * 0.5/1,109,549) of the total production capacity in the economy is destroyed. Subsequently, we examine how the sum of the value added—or the value of production less the total value of intermediates used for the production—of all firms in the economy changes over time. For each set of parameter values, we conduct a simulation 30 times and show the results graphically. We use a solid line for the average value added, while dotted lines are their standard deviations.

Since the simulation in this study requires substantial computational power due to the presence of more than one million agents and five million ties, we use a supercomputer and run simulations in parallel to minimize the run time. The parallel execution reduces the consumption of wall time. The simulation code is shared on GitHub to ensure readers can run their own agents and networks. The code provides abundant variations of simulations.

## Simulation results and discussion

### Benchmark result

**Fig 1:**
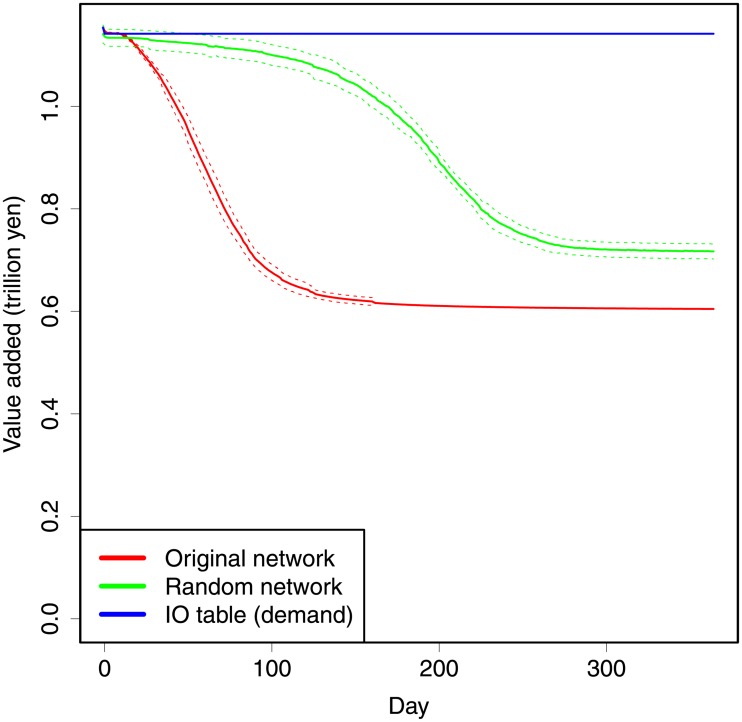
Simulation results for different network structures. The horizontal axis shows days. The vertical axis shows the daily value added of the system. The red lines show the results for the actual network. The green lines show the results for the random networks. The blue line shows the results of the IO table. The solid lines show the average of 30 simulations. The dotted lines show the standard deviations. For all simulations, damages are randomly given to 10,000 firms with 50% production loss (*δ* = 0.5).

In the benchmark test, 50% of the production losses are assigned to 10,000 randomly chosen firms. Our benchmark result using the actual supply chain network and the parameter values explained above is shown using a red line in Fig 1. The simulation result indicates that the value added declines from 1.154 trillion yen per day before the disaster to 1.148 trillion yen per day on day 0. Subsequently, it declines to 1.062 (*σ* = 0.007987) trillion 30 days after the disaster, and then to 0.611 (*σ* = 0.0006345) trillion after 200 days. The numbers with parentheses are standard deviations. After roughly 200 days, it becomes almost stable. Although the direct damage was only 0.5% of the value added per day, the loss of value added reaches approximately 8% on day 30, and then reaches 48% on day 200.

The value added monotonically declines over time and converges to a lower level than the pre-disaster level. The monotonic decreases occur because we do not assume recovery in our model. The recoveries are, for example, restoring production capacity by repair or finding other suppliers or clients. Since we cannot observe the propagation without recovery in the real economy, we only observe it in simulations. Since the simulation reveals the acute decline, we can interpret the simulation results as indicating how negative shocks propagated with speed. Therefore, we conclude that the indirect effects of disasters through supply chain disruptions are significant.

One may wonder why the value added converges to a certain value in the long run. This is because a steady state is achieved when negative shocks reach all firms that are indirectly connected to directly damaged firms in the supply chains.

The recovery is obviously not negligible in the real economy. In the case of the Great East Japan earthquake that occurred in March 2011, firms in the affected areas ceased operations for only five days at the median [24]. On the other hand, Renesas Electronics, a major producer of microcomputers for automobiles, was heavily hit by the earthquake. It recovered three months later in June 2011. Although some factories involved in the final assemblies of automobiles, which were not directly hit, were compelled to stop operation for a few months due to supply chain disruptions, production of automobiles was restored to the pre-earthquake level in July 2011. Therefore, it is important to consider the recovery process in the model if we want to compare the simulation with the real economy. To fill this gap, the model in this study can be expanded to incorporate the recovery. It can replicate the damage propagation and the recovery [21], which simulates the Great East Japan Earthquake and the predicted Nankai Trough Earthquake.

Therefore, the long-run consequences of our simulation may not indicate actual damages from disasters because it ignores recovery processes. However, it is important to know how fast the propagation is because the pace of the propagation should be considered for government intervention. Therefore, when we interpret the simulation results, we focus on the pace of the decline and the total loss in the long run (one year), depending on the context.

### Differences with the random network and the IO table

We show differences in the propagation of negative shocks between the actual network and randomly generated networks. Since massive supply-chain data are normally unavailable, random networks are commonly used in the literature. In fact, the original model, upon which we base our model, used a certain type of random network in its simulation [10].

The degree distribution (the number of links) in the actual supply chain network in Japan is fat-tailed and follows the power law, as found in [25, 26]. It has been repeatedly discussed that networks with power-law distributions, or scale-free networks, show unique properties in many respects [14]. From the viewpoint of supply chains, propagation of negative shocks in the actual network can differ from that in the random networks.

We randomly generate networks with approximately the same number of nodes and links as the actual network (1,109,549 nodes and 5,106,081 links). We thus use the algorithm developed by Gilbert [27]. As mentioned above, the actual and random networks have different degree distributions. In each case, we generate 30 different random networks, and graphically show the average and standard deviation of the change in the value added. In Fig 1, the green lines indicate the results of the random networks. The comparison between the results from the actual and random networks—that is, the red and green lines—clearly show that damages due to indirect effects of the disaster in the short and long run are substantially larger in the actual network than in the random networks. (Here, we use the terms “short run” and “long run” for the first 200 days of the disaster aftermath and the subsequent period, respectively). Thus, losses are likely underestimated if supply chains are assumed to be random, and hence we should use actual networks in practice.

The difference noted earlier can be explained by differences in path lengths in the network. A path length is the number of steps between two arbitrary nodes in a network, and the average path length is the average of all possible path lengths. The average path length of a random network, 〈*d*〉, is proportional to the natural logarithm of the number of nodes, *N*, or 〈*d*〉 ≈ ln*N*. Conversely, a scale-free network, which is the case with the supply chain network in Japan [25], has different properties from random networks [28]. Particularly, the average path length is proportional to the log of the log of the number of nodes: 〈*d*〉 ≈ lnln*N*. In other words, the actual supply chain network has a much shorter average path length than in a random network, suggesting that shocks spread faster in the former network.

Although the path length is longer in the random network, the damages are propagated into the entire network in the end. Thus, one may think that the difference in the final value added of the system should not exist. However, this difference comes from the rationing policy. (Note that the rationing policy is considered realistic.) As shown in the section “Theoretical Model,” the rationing policy indicates that the demand from affected clients is prioritized over the demand from the final consumer (Demand of the final consumers is not affected by the disaster). Therefore, the demand of the final consumer is relatively postponed, which can help work toward absorbing the supply-driven damages. Importantly, the rationing clients lift production in the successive steps of the supply chains, but the rationing final consumers do not show this effect. Longer path lengths lead to increased absorption of the supply shortage. As a result, damages in the actual network with a shorter average path length are substantially greater than those in random networks with a longer path length.

Another comparison is conducted by using the IO table. We use the same initial damages as our simulations. By solving the inverse matrix, we obtain the damage propagation through the IO table. Here, the damage propagation is considered for the demand side, as conducted generally. The blue line in Fig 1 shows the result. Since the analysis of the IO table does not indicate any temporal transition of the value added, the line is shown horizontally to indicate that the damage occurs on day 0 with no following changes. Evidently, the difference in our simulations is tremendous. This result is natural because the IO table analyses do not consider supply constraints. Since a supply shortage is a key factor that affects production under disaster scenarios, the results are understandable. On the other hand, we can simply use the transposed IO table to ensure that the matrix shows supply-side propagations. Clearly, this result is not significantly different from the one for the demand-side analyses because it is an inter-sectoral analysis that cannot incorporate the supply chain.

### Different intensity of shocks

Next, we experiment with different intensities of shocks, assuming the following three cases: 50,000 firms lose 10% of their production capacity; 10,000 firm lose 50% of their production capacity, as in the benchmark case; and 5,000 firms lose 100% of their production capacity. Damaged firms are randomly selected in each case. The total capacity loss in the economy—that is, product of the number of firms and the ratio of production losses—is the same across the three cases.

**Fig 2:**
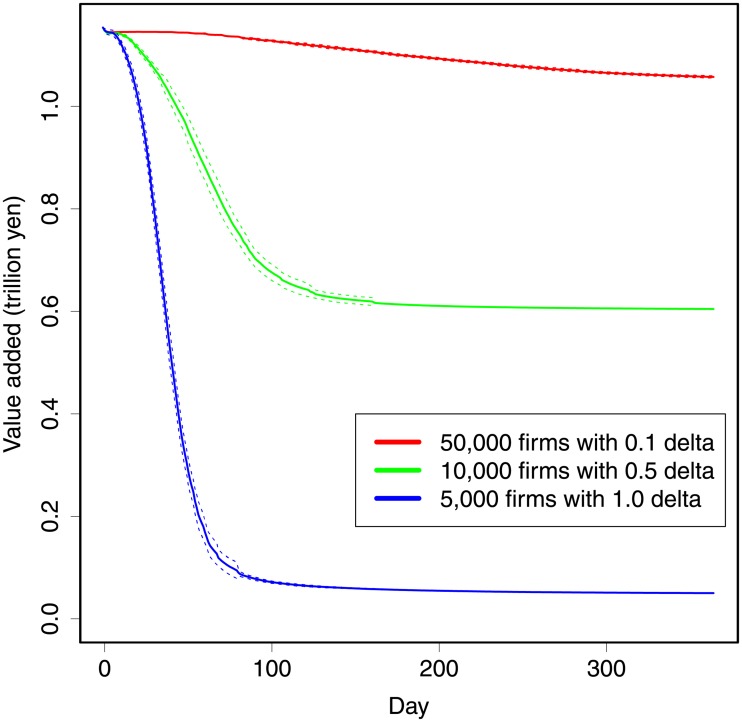
Simulation results for different intensities of shocks. The horizontal axis shows days. The vertical axis shows daily value added of the system. The red lines show the results for 10% production loss (*δ* = 0.1) in 50,000 firms. The green lines show the results for 50% production loss (*δ* = 0.5) in 10,000 firms. The blue lines show the results for 10% production loss (*δ* = 0.1) in 5,000 firms. The solid lines show the average of 30 simulations. The dotted lines show the standard deviations.

The results from the simulation are shown in Fig 2. The different intensities show different speeds of decay; namely, the decay time decreases with the shock intensity. In particular, the most intensive shock shows 80% daily loss in the end.

The result, i.e., the intensive shock shows the shorter decay time, comes from the inventory of firms. As shown in the model, production is constrained by a minimal inventory. Therefore, even if only one supply shows shortage among other plentiful supplies, the firm’s production is limited by this shortage. Hence, the intensive shocks show more serious results than the extensive shocks. The case of Renesas Electronics is an example of this: automobile assemblers had to stop production because of a shortage of microcomputers.

### Substitution among suppliers

Another important issue is substitution of suppliers. It possibly mitigates damages when some intermediate product cannot be provided by a supplier, but other suppliers can provide it. In our model, substitution is realized in Eq (5). Concretely, our model assumes that when the supplier *j* of product *s*, who supplies to firm *i*, is damaged by a disaster, firm *i* can substitute supplies from firm *j* for supplies from firm *k*, which provides the same product *s*. Note that the supply chain is fixed and firm *i* does not find a new supplier. Since the simulation is relatively short (less than one year), this simulation is meaningful. When this kind of substitution is more feasible, indirect damages through supply chains can be mitigated.

To investigate this issue, we experiment with two alternative cases wherein the products (sectors) of suppliers are changed. In one case, the products of firms are randomly shuffled. That is, the distribution of the products is preserved, such as in actual supply chains. Since firms use a variety of intermediates, we expect the substitution to be more difficult compared with actual supply chains. In another alternative case, each firm has a different product. Therefore, there is no substitution. In economics literature, substitutions occur between different products by introducing elasticity. However, in our simulations, we only consider substitutions between the same products from different suppliers.

**Fig 3:**
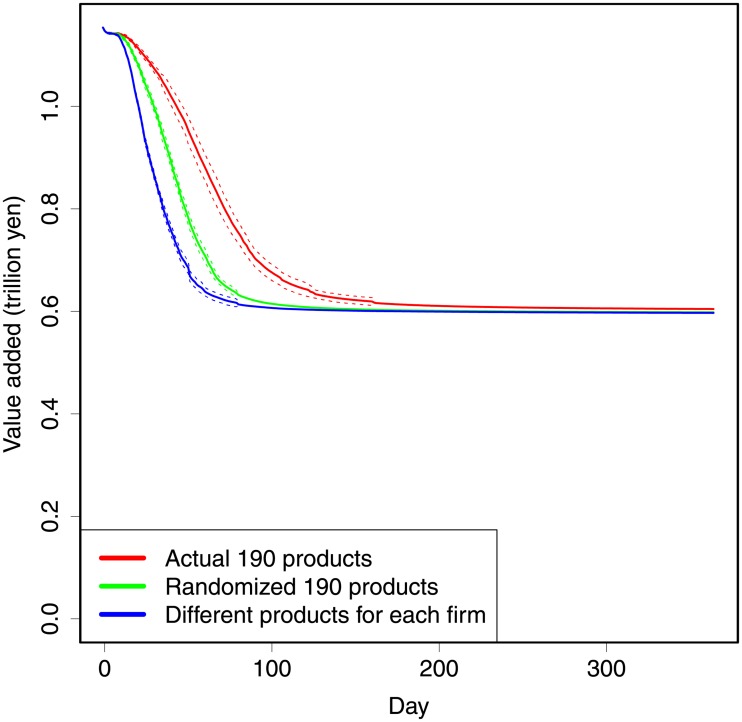
Simulation results to show effect of substitution. The horizontal axis shows days. The vertical axis shows daily value added of the system. The red lines show the results for the actual network. The green lines show the results for the product-randomized networks. The blue lines show the results for no substitution. The solid lines show the average of 30 simulations. The dotted lines show the standard deviations. For all simulations, damages are randomly assigned to 10,000 firms that face 50% production loss (*δ* = 0.5).

Fig 3 shows the results by using the benchmark case (the red line) and by assuming a random assignment of products (green) and completely differentiated products (blue). This figure indicates that the propagation of negative shocks is substantially faster in the latter two cases wherein substitution is more difficult than in the benchmark case. Therefore, the benchmark case, that is, actual network, substitutes supplies as part of its innate structure. Therefore, substitution is an important channel for mitigating shock propagation in actual supply chains. Especially, we can see the innate resistance in the comparison between the benchmark and the random assignment.

### Regional and sectoral damages

So far, we assumed that firms affected by the disaster are randomly selected across regions and sectors. However, if we consider natural disasters, such as earthquakes and typhoons, they affect specific regions. Additionally, shocks in the economy may be caused not only by natural disasters but also by financial crises or trade sanctions. These human-made shocks are more likely to be sector-specific. Therefore, we now examine propagation of regional and sectoral shocks.

**Fig 4:**
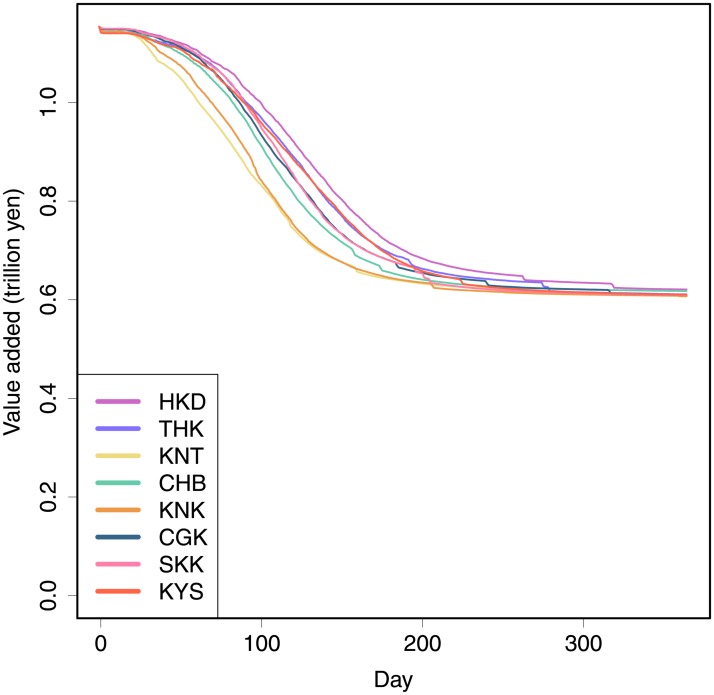
Simulation results for different regional shocks. The horizontal axis shows days, whereas the vertical axis shows daily value added of the system. The solid lines show the average of 30 simulations. It is assumed that 10,000 firms randomly chosen in each of the eight regions—Hokkaido (HKD), Tohoku (THK), Kanto (KNT), Chubu (CHB), Kinki (KNK), Chugoku (CGK), Shikoku (SKK), and Kyusyu (KYS)—face a 50% loss in production capacity. Standard deviations are omitted for visibility.

First, we assume that firms in a particular region are damaged. We divide Japan into eight regions—Hokkaido, Tohoku, Kanto, Chubu, Kinki, Chugoku, Shikoku, and Kyusyu. As in the benchmark simulation, we assume that the 10,000 firms randomly chosen from each region face a 50% loss in production capacity. Results in Fig 4 show that damages in Kanto and Kinki propagate most rapidly, although the total loss in the long run is the same across regional damages. Kanto includes the largest metropolitan area in Japan, such as Tokyo and Yokohama, whereas Kinki includes the second, Osaka. Therefore, when compared with the propagation in remote areas, our results indicate that damages propagate faster when industrial areas are hit. Rapid propagation implies significant damages in the short run. The Great East Japan Earthquake in 2011 hit the Tohoku region, which is less industrialized. On the other hand, the speculation by the Japanese government indicates that a predicted great earthquake, such as the Tokyo Inland Earthquake and the Nankai Trough Earthquake, will hit Kanto or Kinki regions; these regions show faster propagations in the simulations. Therefore, the loss caused by the disasters will be substantially larger than the earthquake in 2011.

**Fig 5:**
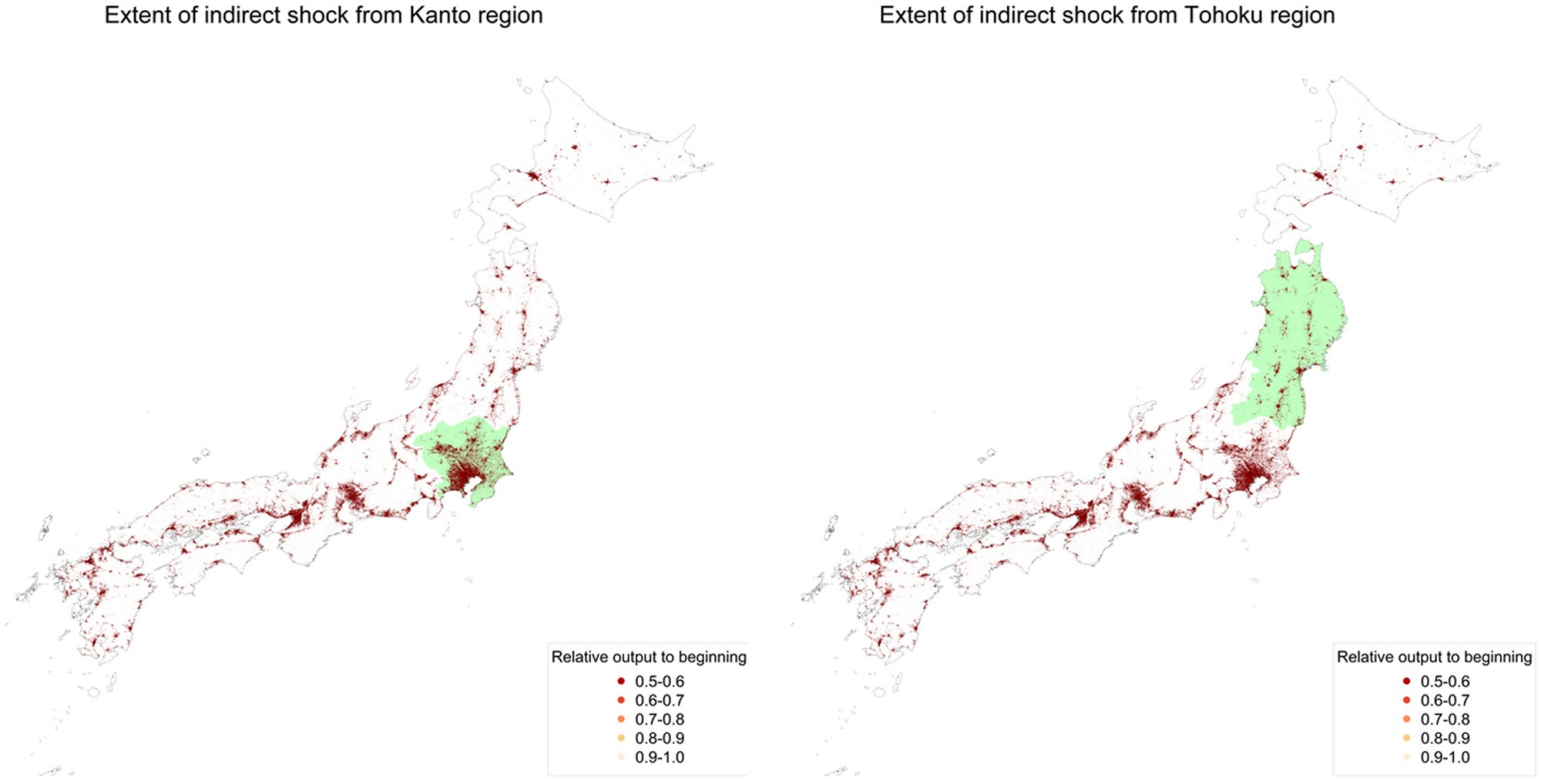
Geographical plots of regional shocks. The left figure shows a snapshot of relative output for the beginning of day 30 in Kanto region, colored in light green. The right figure corresponds to the same simulation for Tohoku region.

On the other hand, the results do not show significant difference in long-run damages between regional shocks. This is because most firms are connected through supply chains. To understand this, Fig 5 illustrates damages on day 30 in the two cases, one in which the Kanto region—the economic center of Japan that includes Tokyo—is directly hit, and the other in which the Tohoku region—a relatively less developed region—is hit. The left and right parts of the figure show the geographical plots of affected firms due to direct damages in Kanto and Tohoku, respectively. In both cases, damages reach most regions of Japan in 30 days. Even in the 30 days, the damages already spread and we do not see an explicit difference. Thus, only immediate intervention, such as governmental aid, can be effective because the damage propagates faster into the entire economy through supply chains.

**Fig 6:**
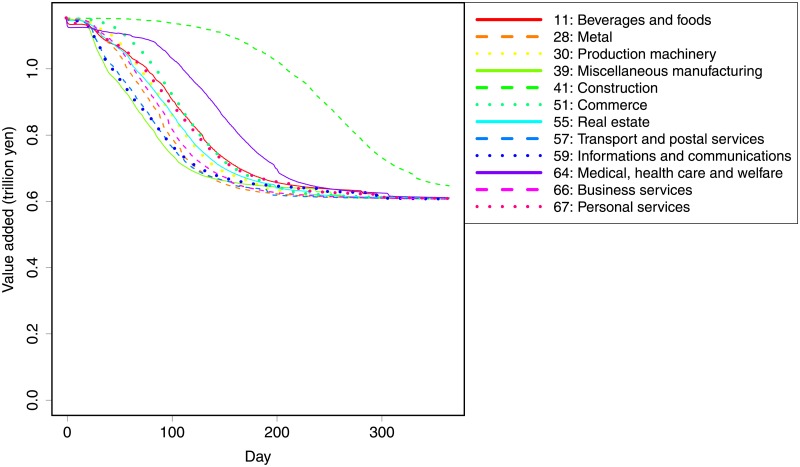
Simulation results for different sectoral shocks. The horizontal axis shows days, whereas the vertical axis shows daily value added of the system. The solid lines show the average of 30 simulations. It is assumed that 10,000 firms randomly chosen from each of 12 sectors face a 50% loss in production capacity. Standard deviations are omitted for visibility.

Second, we assume that firms in a particular sector, among the 190 sectors, are damaged. Since we need more than 10,000 firms in a sector to run the simulation, we focus on 12 sectors with more than 10,000 firms in our data. The list of the 12 sectors and the simulation results are shown in Fig 6. The results indicate substantial variations in the short run—the propagation is faster when some sectors, such as miscellaneous manufacturing, information and communications, and transport and postal services, are directly damaged, while it is slow when others, such as real estate and medical, healthcare and welfare, are affected. Most notably, when the construction sector is hit, there is little propagation of negative shocks.

The results can be interpreted by considering whether hub firms exist in the directly damaged sector. A scale-free network has a few hubs that have numerous links. Therefore, if the sector includes such hubs or includes firms connected to hubs in short steps, then the propagation would be fast. Therefore, the construction sector records an extremely slow decline as there are numerous non-hub firms and they form many layers of closed supply chains. Therefore, the shocks are slow to propagate in this sector.

### Single-firm damages

It is beneficial to know how a single-firm loss will affect the entire economy, that is, a single firm may cause substantial damage to the entire economy. This is because a single firm can easily shutdown as a result of bankruptcy and suspension, among other reasons. Intuitively, it seems that single-firm loss cannot seriously impact the entire economy, but it is not the case, as shown below.

In a simulation, only one firm is completely destroyed at once. The influence of the simulation is calculated using the following equation: (the summation of lost value added in a year) / (the summation of value added without damages in a year). Henceforth, we will call this “system damage.” Theoretically, the system damage can take a value from 0 to 1. Note that firms experience no recovery in the simulations. Therefore, the indirect damage monotonously expands as days proceed.

**Fig 7:**
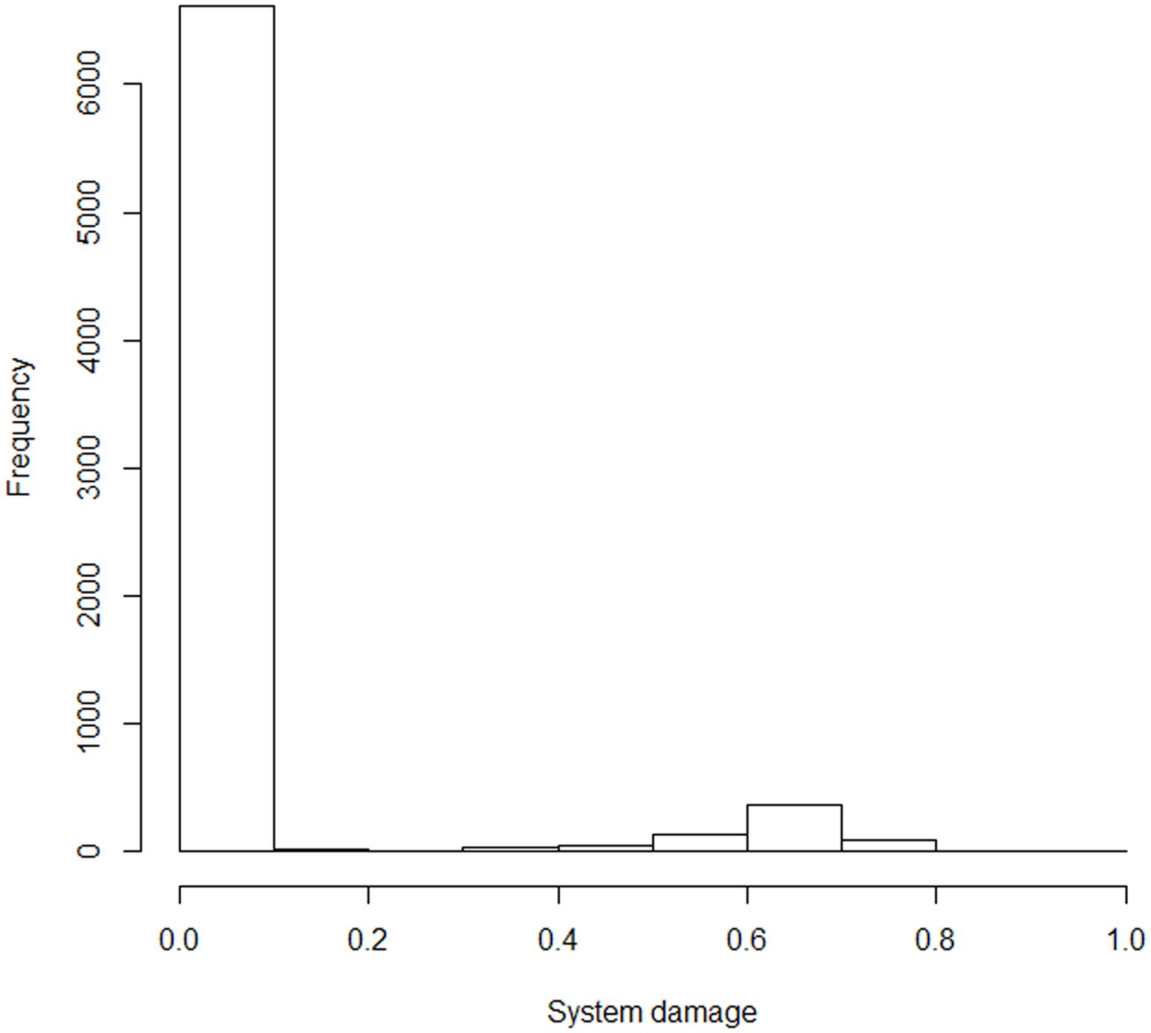
Histogram of system damage: Horizontal axis is delimited by 0.1.

We simulate 7,332 cases with randomly chosen firms. All firms are not tested because of a limitation in computational resources. Fig 7 shows the histogram of the system damage. The result shows that approximately 90% of firms show system damage of less than 0.1. Precisely, 86.6% of the sampled firms experience less than 10^−5^ system damage. Conversely, 9.7% firms cause serious system damage. These firms incur a value of more than 0.1.

If we consider the small-world property of the network and no recovery of firms, it can be said that the supply chain has strong robustness. That is, if the failure is random, the systemic risk is not large. On the other hand, the economic system is vulnerable against selective attacks.

We check the Kendall correlation coefficients between the system damage and attributes of firms. The attributes are degree (number of suppliers and clients), in-degree (number of suppliers), out-degree (number of clients), amount of labor, number of institutions, number of factories, sales, and capital. The degree shows 0.326 as the correlation coefficient, which is the largest, and the out-degree shows 0.325, which is the second largest. In contrast, the in-degree shows 0.239.

The results show that the number of suppliers and clients is important for ascertaining risk of single firm failure, among other attributes. Since the out-degree has a larger coefficient, the number of clients, than the number of suppliers, matters more in terms of systemic risk. This finding indicates that the downstream shock is more serious than the upstream shock.

## Conclusion

We used Japanese nation-wide supply-chain network data and employed a modified version of Hallegatte’s model [10] to examine how negative shocks by artificial disasters propagate through supply chains. We obtained the following results. First, network structures severely affect the speed of propagation in the short run and the total loss in the long run. The scale-free nature of the actual supply-chain network, that is, the power-law degree distribution, leads to faster propagation than the random network. Second, a small number of firms with intense damages cause faster and larger propagation. Third, substitution among suppliers largely contributes to economic resistance. The pace of the propagation of negative shocks increases with an increase in substitution difficulties. Fourth, direct damages in industrial regions promote faster propagation than those in less industrial regions, although the total loss in value added in the long run is the same. Fifth, different sectoral damages cause large differences in the speed of propagation. Particularly, the effects of direct damages on the construction sector are quite small. Finally, an estimation of the indirect damage triggered by a single-firm loss shows that 86.6% of firms cause less than 10^−5^ of the damage to the entire economy. On the other hand, 9.7% of firms cause more than 10% of the damage to the entire supply chain. Thus, the actual supply chain has strong robustness against random failures, but it is vulnerable to selective attacks.

These results imply that we cannot use only the size of direct damages by negative shocks to predict the outcome of the economic loss. Adversely, different initial damages can generate considerably different damages, depending on the property of the supply chain network in the economy.

We note the limitations of this study and future work addressing them. First, the setting of some parameters was not validated. The parameters are the target inventory size and the daily ratio to adjust the inventory size. The parameters can be directly set based on the observation, or calibrated through simulations. The calibration will be discussed in the upcoming work as a continuation of this study [21]. Second, since the initial shocks in this study are artificial and there is no recovery from shocks, a comparison with real shocks was not discussed. This is also addressed in the upcoming work [21]. Finally, we can expand the model in terms of the price and equilibrium mechanism. In fact, a preceding paper has discussed the artificial shocks on global supply chains [29], which is based on the equilibrium mechanism and the agent based model. Furthermore, by considering the price, we can consider the autonomous choice of trade partners, which generates a more realistic model than a model with a fixed network structure [30].
